# Supplementary material for: Utility of smart watches for identifying arrhythmias in children
Source: Commun Med (Lond). 2023 Dec 13;3:167. doi: 10.1038/s43856-023-00392-9 (PMC10719318; doi:10.1038/s43856-023-00392-9)
Supplement: Supplementary file 2 — Reporting Summary [file 43856_2023_392_MOESM2_ESM.pdf]

nature portfolio

Corresponding author(s): Aydin Zahedivash, MD, MBA

Last updated by author(s): Aug 1, 2023

Reporting Summary

Nature Portfolio wishes to improve the reproducibility of the work that we publish. This form provides structure for consistency and transparency in reporting. For further information on Nature Portfolio policies, see our Editorial Policies and the Editorial Policy Checklist.

Statistics

For all statistical analyses, confirm that the following items are present in the figure legend, table legend, main text, or Methods section.

n/a	Confirmed	
☐	☒	The exact sample size (*n*) for each experimental group/condition, given as a discrete number and unit of measurement
☐	☒	A statement on whether measurements were taken from distinct samples or whether the same sample was measured repeatedly
☒	☐	The statistical test(s) used AND whether they are one- or two-sided<br>*Only common tests should be described solely by name; describe more complex techniques in the Methods section.*
☐	☒	A description of all covariates tested
☐	☒	A description of any assumptions or corrections, such as tests of normality and adjustment for multiple comparisons
☐	☒	A full description of the statistical parameters including central tendency (e.g. means) or other basic estimates (e.g. regression coefficient) AND variation (e.g. standard deviation) or associated estimates of uncertainty (e.g. confidence intervals)
☒	☐	For null hypothesis testing, the test statistic (e.g. *F*, *t*, *r*) with confidence intervals, effect sizes, degrees of freedom and *P* value noted<br>*Give P values as exact values whenever suitable.*
☒	☐	For Bayesian analysis, information on the choice of priors and Markov chain Monte Carlo settings
☒	☐	For hierarchical and complex designs, identification of the appropriate level for tests and full reporting of outcomes
☒	☐	Estimates of effect sizes (e.g. Cohen's *d*, Pearson's *r*), indicating how they were calculated

Our web collection on statistics for biologists contains articles on many of the points above.

Software and code

Policy information about availability of computer code

Data collection	N/A
Data analysis	All numerical analysis was done using STATA version 15 (College Station, TX).

For manuscripts utilizing custom algorithms or software that are central to the research but not yet described in published literature, software must be made available to editors and reviewers. We strongly encourage code deposition in a community repository (e.g. GitHub). See the Nature Portfolio guidelines for submitting code & software for further information.

Data

Policy information about availability of data

All manuscripts must include a data availability statement. This statement should provide the following information, where applicable:

- Accession codes, unique identifiers, or web links for publicly available datasets
- A description of any restrictions on data availability
- For clinical datasets or third party data, please ensure that the statement adheres to our policy

Since the data used in this study involves medical records, the datasets generated and analyzed during the current study are not publicly available to protect patient privacy but are available from the corresponding author on reasonable request.

Human research participants

Policy information about studies involving human research participants and Sex and Gender in Research.

Reporting on sex and gender	Sex and gender was not a central component of our study, and persons of all identities were included in our retrospective study through our database query for patients with arrhythmia who had any documented mention of the words "Apple Watch" in clinical records. Distribution of assigned sex of our entire sample based on self-reporting in the electronic health record is noted in Table 1.
Population characteristics	This study investigated the characterization of cardiac arrhythmias in children in particular. Details on the diagnoses and underlying diseases are included in Tables 1-3 and are listed in the results section of the manuscript.
Recruitment	This was a single-center, retrospective analysis performed for all patients ≤18 years of age who had signs of an arrhythmia documented by an Apple Watch between 2018 and 2022 and received a formal arrhythmia diagnosis at Lucille Packard Children's Hospital Stanford. In determining the patient sample, patient documentation was queried for any mention of the key phrase "Apple Watch". All patients with an arrhythmia captured by their Apple Watch with an arrhythmia confirmation by a pediatric electrophysiologist were included in the analysis.<br><br>The cohort of patients in this analysis are also from a large referral center, and there is likely some degree of selection bias, with a higher degree of patients referred to our center with true arrhythmias detected. Additionally, our study involved patients who had access to a personally owned Apple Watch, biasing our sample to those who would have the interest and the means to obtain and use such a device. Both of these potential sources of bias are noted in the limitations section of our manuscript.
Ethics oversight	The Stanford University Institutional Review Board approved our study. This is listed in the manuscript.

Note that full information on the approval of the study protocol must also be provided in the manuscript.

Field-specific reporting

Please select the one below that is the best fit for your research. If you are not sure, read the appropriate sections before making your selection.

☒ Life sciences ☐ Behavioural & social sciences ☐ Ecological, evolutionary & environmental sciences

For a reference copy of the document with all sections, see nature.com/documents/nr-reporting-summary-flat.pdf

Life sciences study design

All studies must disclose on these points even when the disclosure is negative.

Sample size	As mentioned above, this was a single-center, retrospective analysis performed for all patients ≤18 years of age who had signs of an arrhythmia documented by an Apple Watch between 2018 and 2022 and received a formal arrhythmia diagnosis at Lucille Packard Children's Hospital Stanford. In determining the patient sample, patient documentation was queried for any mention of the key phrase "Apple Watch". All patients with an arrhythmia captured by their Apple Watch with an arrhythmia confirmation by a pediatric electrophysiologist were included in the analysis. No specific calculation was made to determine sample size, rather, we extended our retrospective chart review to the earliest year that the ECG feature was made available for the Apple Watch (2018).
Data exclusions	Any data that met the inclusion criteria above were included. No patients were excluded.
Replication	All data was collected and analyzed by two independent physician scientists.
Randomization	Randomization was not necessary in this retrospective chart review.
Blinding	Blinding was not possible in this study as it was a retrospective chart review which necessitated access to patient identifying information to co-register clinical data with Apple Watch findings. After data was collected and codified, however, analysis was done with de-identified data.

Reporting for specific materials, systems and methods

We require information from authors about some types of materials, experimental systems and methods used in many studies. Here, indicate whether each material, system or method listed is relevant to your study. If you are not sure if a list item applies to your research, read the appropriate section before selecting a response.

Materials & experimental systems

n/a	Involved in the study
☒	☐ Antibodies
☒	☐ Eukaryotic cell lines
☒	☐ Palaeontology and archaeology
☒	☐ Animals and other organisms
☐	☒ Clinical data
☒	☐ Dual use research of concern

Methods

n/a	Involved in the study
☒	☐ ChIP-seq
☒	☐ Flow cytometry
☒	☐ MRI-based neuroimaging

Clinical data

Policy information about clinical studies

All manuscripts should comply with the ICMJE guidelines for publication of clinical research and a completed CONSORT checklist must be included with all submissions.

Clinical trial registration

N/A - this is a single-center retrospective study

Study protocol

N/A - see methods section of the manuscript

Data collection

As noted in the manuscript, this is a single center, retrospective analysis performed for all patients ≤18 years of age who had signs of an arrhythmia documented by an Apple Watch between 2018 and 2022 and received a formal arrhythmia diagnosis. All data was collected at the Lucile Packard Children's Hospital Stanford. The role that the Apple Watch had in arrhythmia diagnosis, the results of other ambulatory cardiac monitoring studies, and findings of any EP studies were evaluated.

Outcomes

Primary outcome: Method of arrhythmia characterization using the Apple Watch. We selected this as our primary outcome as it allowed us to best understand how the passive and active monitoring features of the Apple Watch were being used to characterize arrhythmia events in children, and provided insight into the potential use cases for wearables in pediatric arrhythmia diagnosis. We assessed for this outcome by analyzing the clinical documentation and Apple Watch data associated with each patient's arrhythmia to determine the method of arrhythmia detection.

Secondary outcome: Whether the Apple Watch was able to capture an arrhythmia event that traditional ambulatory monitoring devices could not detect. We chose this as a secondary outcome because it helped us further understand the passive monitoring capabilities of the Apple Watch in pediatric arrhythmia workup. We were further able to delineate how often the ECG vs heart rate monitoring features were used to capture the arrhythmias. We assessed for this outcome by comparing Apple Watch results during arrhythmia episodes with results of any ambulatory monitoring studies that the patient underwent during the diagnostic workup period.

Secondary outcome: Whether the Apple Watch contributed to a new arrhythmia diagnosis. We chose this as an important secondary outcome because it gave insight into how the Apple Watch was being used in clinical care--either as a tool to aid in a new diagnosis or as one that confirmed a preexisting diagnosis. We assessed for this secondary outcome by examining when in the sequence of clinical care and diagnostic workup that the Apple Watch was incorporated into the patient's care.

Secondary Outcome: Types of arrhythmias that the Apple Watch was able to characterize. We chose this as a secondary aim so we could better assess the clinical relevance of a wearable device such as the Apple Watch to characterize the arrhythmias which are most common in children. We assessed for this secondary outcome by comparing the Apple Watch data to current ambulatory gold standards and any available invasive electrophysiology studies.
